# Quantitative Comparison of the Walk and Trot of Border Collies and Labrador Retrievers, Breeds with Different Performance Requirements

**DOI:** 10.1371/journal.pone.0145396

**Published:** 2015-12-21

**Authors:** Brittany Jean Carr, Sherman O Canapp, M. Christine Zink

**Affiliations:** 1 Veterinary Orthopedic and Sports Medicine, 10975 Guilford Rd., Annapolis Junction, MD, 20701, United States of America; 2 Zink Integrative Sports Medicine, 12701 Folly Quarter Rd., Ellicott City, MD, 21042, United States of America; University of Florida, UNITED STATES

## Abstract

**Introduction:**

We hypothesized that breed differences of Border Collies and Labrador Retrievers would be reflected in the temporospatial characteristics of the walk and trot.

**Materials and Methods:**

Twenty healthy Border Collies and 20 healthy Labrador Retrievers made three passes across a pressure sensing walkway system that recorded quantitative temporospatial information at a walk and a trot. The following variables were measured for each dog: velocity, total pressure index percentage (TPI%), ratio of weight borne on the thoracic vs. pelvic limbs (T/P TPI%), stance time percentage (ST%), and thoracic limb stride length (TSrL).

**Results:**

The mean T/P TPI% for Border Collies at a walk and at a trot were significantly lower than for Labrador Retrievers (*p* = 0.0007 and *p* = 0.0003). Border Collies had a significantly lower ST% than Labrador Retrievers for the thoracic limbs and pelvic limbs at a walk (*p* = 0.0058 and 0.0003) and the trot (*p* = 0.0280 and 0.0448). There was no relationship between ST% and TSrL in Border Collies and an inverse correlation between ST% and TSrL in Labrador Retrievers (*p* = 0.0002).

**Discussion:**

Key quantitative gait differences were identified in Border Collies and Labrador Retrievers, which could potentially provide each breed with an advantage for their working function.

## Introduction

Border Collies and Labrador Retrievers are two popular breeds used for a variety of competitive performance events. These breeds have evolved to have structural characteristics that were empirically advantageous for their different functions. Border Collies were bred to fetch and herd sheep that are scattered over hundreds of acres, requiring them to be extremely agile, turn sharply and move quickly to keep the flock together regardless of the erratic behavior of the sheep or changes in topology of the land. The Labrador Retriever was bred to fetch upland waterfowl, which might range in size from small quail to large geese. To be successful, they must run straight to the game, recover it, and return directly to the hunter, regardless of the vegetation [1,2]. We hypothesized that these two breeds would have differences in the temporospatial characteristics of the walk and trot.

Gait analysis is an important diagnostic tool not only to establish a diagnosis but also to monitor treatment efficacy and determine when the dog has achieved full recovery after injury. Understanding normal gait and individual breed characteristics is critical in diagnosing dogs with orthopedic conditions and identifying early abnormalities prior to frank lameness. Discrepancies have been found between numerical rating scales, visual analogue scoring scales, and force plate gait analysis [3]. Recent studies showed that dogs with undiagnosed cranial cruciate ligament rupture could not be distinguished from clinically normal dogs on the basis of peak vertical force alone as measured by force plate. Hence, a multivariate approach to lameness evaluation was suggested [3–5].

Pressure-sensing walkways have been validated to analyze normal and abnormal gaits in dogs and aid in diagnosing orthopedic, muscular, and neurological disorders that affect gait [6–12]. Light et al previously established a protocol for temporal-spatial gait analysis using a portable walkway system in healthy Labrador Retrievers at a walk [6]. The walkway system records temporospatial variables, including total pressure index percentage (TPI%) for the each limb from which the ratio of weight borne on the thoracic vs. pelvic limbs (T/P TPI%) can be calculated, as well as stance time percentage (ST%) for each limb, and stride length of each limb (SrL).

Studies have compared the gait of the Labrador Retriever to the Rottweiler and the Greyhound and found differences in ground reaction forces [13–16]. These differences were attributed to variation in conformation and body weight. These studies suggest that each breed should have a breed-specific database and reference ranges for gait variables [17–21]. To the best of our knowledge, there are no published studies comparing the gait characteristics of breeds of dogs with very different original functions that require differing types of movements. We hypothesized that breed differences of Border Collies and Labrador Retrievers would be reflected in differences in gait characteristics as measured by a pressure sensing walkway. Twenty healthy Border Collies and 20 healthy Labrador Retrievers made three passes across a pressure sensing walkway system that recorded quantitative temporospatial information at a walk and a trot. The following variables were measured for each dog: velocity, total pressure index percentage (TPI%), ratio of weight borne on the thoracic vs. pelvic limbs (T/P TPI%), stance time percentage (ST%), and thoracic limb stride length (TSrL).

## Methods and Materials

### Ethics Statement

In accordance with AAALAC International Rules of Accreditation, this study was performed with the approval of the VOSM Research Committee and with owner consent. In this study, all dogs who participated were client-owned dogs deemed healthy by a veterinarian. All clients volunteered their dog for the study and provided written consent as required by Veterinary Orthopedic and Sports Medicine Group for every study participant. No therapies, treatments, or interventions were administered to any dogs included in the study. All dogs that participated in the study were directly overseen by a veterinarian to ensure no harm was incurred during study participation, and the owners were present at all times during the evaluation of the dogs.

### Study Dogs

Twenty healthy Border Collies and twenty healthy Labrador Retrievers were included in this study. There were 7 intact females (4 Border Collie, 3 Labrador Retriever), 10 spayed females (4 Border Collie, 6 Labrador Retriever), 15 intact males (9 Border Collie, 6 Labrador Retriever), and 8 neutered males (3 Border Collie, 5 Labrador Retriever). Of the 20 Border Collies, 1 was a companion dog and 19 were performance dogs competing in the following sports: 10 agility, 1 flyball, 2 obedience, and 12 herding. Of the 20 Labrador Retrievers, 3 were companion dogs and 17 were performance dogs, competing in the following sports: 10 agility, 9 rally, 1 flyball, 7 obedience, 3 conformation, 2 field trials, 5 hunt tests, 2 dock diving, 1 weight pulling, and 1 tracking.

All dogs were client-owned and recruited via an advertisement placed on a clinic website and in social media. Inclusion criteria for this study were that the patient must be healthy and orthopedically sound with no previous history of injury or orthopedic disease. To ensure that all dogs were orthopedically sound, a complete orthopedic examination was performed on each dog. All major muscle masses, bones, and joints were thoroughly examined. Each joint was evaluated for range of motion with goniometry, and palpated for crepitus, effusion, pain, and instability. Forelimb and pelvic limb circumferences were also measured. Any patient with an abnormality detected upon orthopedic examination was excluded from the study. Further, if lameness was detected on gait analysis, the patient was excluded from the study. In addition to orthopedic examination findings, the patient’s body weight (BW), body condition score (BCS), and height at the withers at a stance (W; measured as the distance from the ground to the top of the scapula) were also recorded.

### Gait Analysis System

Objective gait analysis was performed in a quiet room using a temporospatial pressure sensing walkway. The walkway system was equipped with a 8.23 x 0.85-m portable mat containing 29,952 encapsulated sensors (GaitFour Pressure Sensing Walkway, platinum version, CIR Systems Inc, Harvertown, PA). The active dimensions of the mat were 8.04 x 0.61-m. A 1.25 x 0.85-m section of inactive mat was placed at each end of the walkway system to provide transition surfaces when entering and exiting the system. The mat was calibrated by the manufacturer. The walkway system interfaced with a computer and software program for processing and storage of raw data recorded from quadruped gait analysis (GaitFour, platinum version, CIR Systems Inc, Harvertown, PA). One camera was positioned at a height of 50 cm at the end of the walkway system to record movement (Logitech MP web camera, Logitech, Freemont, CA). Digital video files of each pass across the walkway system were automatically linked to the data files for footfall verification.

Every dog was handled by the same examiner and allowed to acclimate to the room prior to data collection. Dogs were walked on the mat until they appeared relaxed (approximately 4 to 6 passes/dog). Three passes across the walkway were recorded at both the walk and the trot. A pass was defined as a dog moving along the length of the portable walkway system in one direction and consisted of 4 to 6 gait cycles. Inclusion criteria for a pass were that the dog was at a walk (velocity, 0.9 to 1.2 m/s) or a trot (velocity, 1.8 to 2.6 m/s), had minimal head turning, and that gait cycle velocities did not vary by more than 10%. The first 3 passes that met the inclusion criteria were analyzed for each dog. Videos of each pass were reviewed to ensure all inclusion criteria were met.

The software program was used to identify the paw print of each footfall. The software program then performed analysis of multiple variables for each pass. Data gathered or calculated from each pass provided the following mean values: velocity, total pressure index percentage for each foot (TPI%; the sum of peak pressure values recorded from each activated sensor by a paw during mat contact/total sum of peak pressure values for all feet x 100), thoracic to pelvic limb TPI% (T/P TPI%; sum of peak pressure values recorded from the thoracic limbs/sum of peak pressure values recorded from the pelvic limbs x 100), stance time percentage for thoracic and pelvic limbs (ST%; the proportion of stance time of the each limb to total gait cycle time), stride length for each limb (SrL; the distance between 2 successive strikes of the same paw), step length for the thoracic and pelvic limbs (StL; the distance between the caudal point of the central pad of one foot to the same location on the central pad of the contralateral foot).

### Statistical Analysis

Gait characteristics were analyzed using statistical software (GraphPad Prism 6, GraphPad Software Inc, La Jolla, CA). All data were tested for normality and found to have a normal distribution. Therefore, groups of data were compared using unpaired 2-tailed Student’s *t*-tests. Correlations between groups of data were analyzed using Pearson’s tests. Significance was established at *p* = 0.05.

## Results

**Fig 1:**
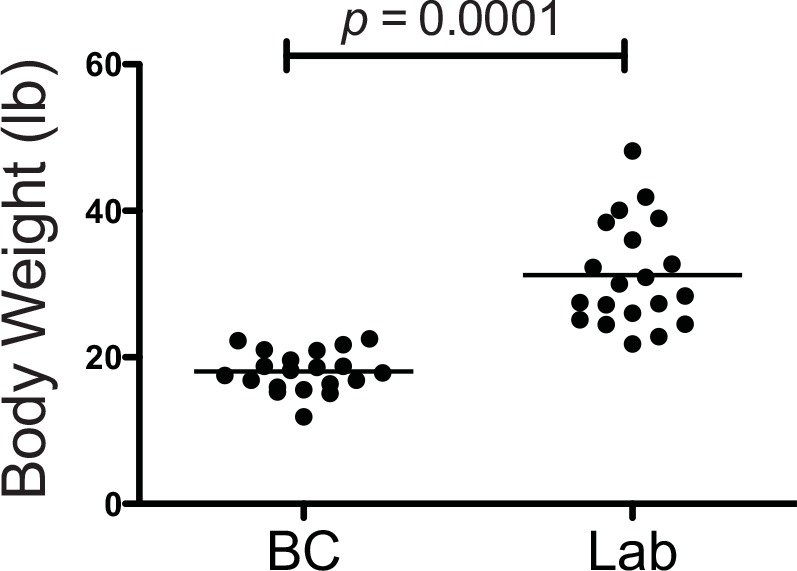
Body Weights of Border Collies and Labrador Retrievers. The Border Collies in this study weighed significantly less than the Labrador Retrievers.

The ages of the Border Collies (mean 3.6 years; range 1 to 7 years) was not significantly different from those of the Labrador Retrievers (mean 4.1 years; range 1 to 11 years). The Border Collies had significantly lower body condition scores (BCS) than the Labrador Retrievers (mean BCS 4.3 vs 5.4, respectively; σ = 0.5 vs σ = 1, respectively; *p* = 0.0007) and weighed significantly less on average (mean weight 18.1 kg vs mean weight 31.2 kg; σ = 2.7 kg vs σ = 7.2 kg, respectively; *p* = 0.0001; Fig 1).

The Border Collies traveled at a mean velocity of 1.32 m/s at the walk and 2.32 m/s at the trot, while the Labrador Retrievers traveled at a mean velocity of 1.16 m/s at the walk and 2.27 m/s at the trot. Differences in velocity for the same gait were not significant between the two breeds.

**Fig 2:**
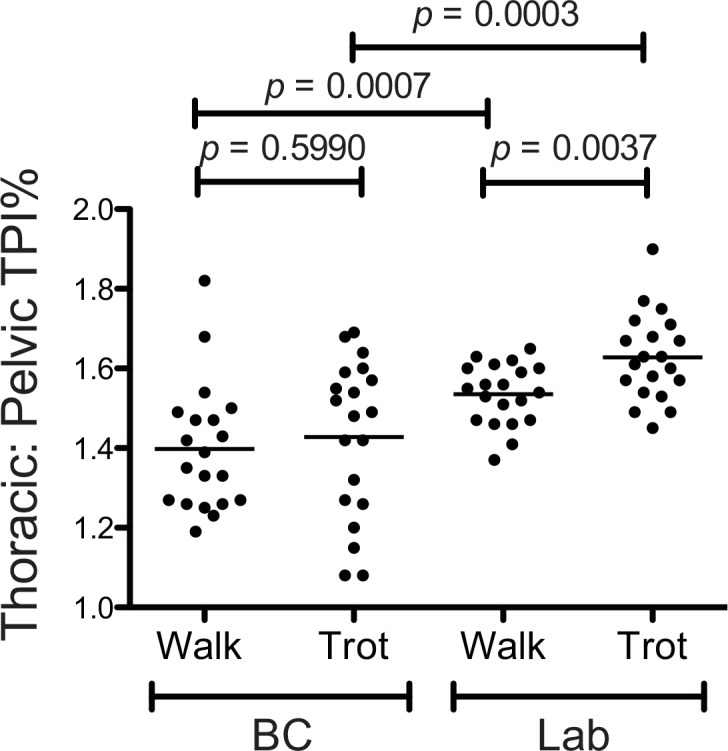
Thoracic to Pelvic Limb Total Pressure Index Percentage Ratio (T/P TPI%) at the Walk and Trot in Border Collies and Labrador Retrievers. The Border Collies had significantly lower ratios of weight borne on the thoracic vs the pelvic limbs (T/P TPI%) than the Labrador Retrievers at the walk (p = 0.0007) and at the trot (p = 0.0003). There was no difference in the relative weight borne on the thoracic vs. pelvic limbs of the Border Collies at the walk and trot (p = 0.5990), whereas the Labrador Retrievers bore more weight on the thoracic vs. pelvic limbs at the trot as compared to the walk (p = 0.0037).

The Mean Total Pressure Index Percentage (TPI%) at the walk and trot for the Border Collies and Labrador Retrievers were recorded (Table 1)**.** When evaluating the Total Pressure Index Percentage (TPI%) at the walk, the average TPI% of the thoracic and pelvic limbs for the Border Collies were 58.3% and 42.0%, respectively. Thus, the average ratio of weight borne by the Border Collies on the thoracic vs. pelvic limbs (T/P TPI%) at the walk was 1.40 (Fig 2). The average TPI% of the thoracic and pelvic limbs for the Labrador Retrievers at the walk were 60.5% and 39.5%, respectively, resulting in a T/P TPI% of 1.53. The T/P TPI% for the Border Collies at the walk was significantly lower than for the Labrador Retrievers (*p* = 0.0007).

**Table 1:** Mean Total Pressure Index Percentage (TPI%) at the Walk and Trot for Border Collies and Labrador Retrievers.

	Mean Left Front TPI	Mean Right Front TPI	Mean Left Hind TPI	Mean Right Hind TPI
Border Collie Walk	25.3 +/- 4.9	25.5 +/- 5.1	18.1 +/- 3.9	18.4 +/- 3.8
Border Collie Trot	32.9 +/- 7.1	33.4 +/- 6.9	23.2 +/- 5.1	23.9 +/- 5.6
Labrador Retriever Walk	43.0 +/- 9.4	43.1 +/- 10.2	28.4 +/- 6.5	27.9 +/- 6.2
Labrador Retriever Trot	61.1 +/- 10.4	60.7 +/- 10.5	37.85 +/- 8.0	37.9 +/- 8.1

While at the trot, the average TPI% of the thoracic and pelvic limbs for the Border Collies were 58.4% and 41.3%, respectively. The T/P TPI% for Border Collies was thus 1.41 (Fig 2). The average TPI% of the thoracic and pelvic limbs for the Labrador Retrievers at the trot were 61.9% and 38.1%, respectively. The T/P TPI% for the Labrador Retrievers was thus 1.62. The ratios of weight borne on the thoracic vs the pelvic limbs of the Border Collies at the trot was significantly lower than for the Labrador Retrievers (*p* = 0.0003).

The T/P TPI% at the walk and the trot were compared for both breeds. For the Border Collies, there was no significant difference in the T/P TPI% at the walk as compared to the trot *(p* = 0.5990*)*. However, the Labrador Retrievers had significantly higher T/P TPI% at the trot than at the walk (*p* = 0.0037).

**Fig 3:**
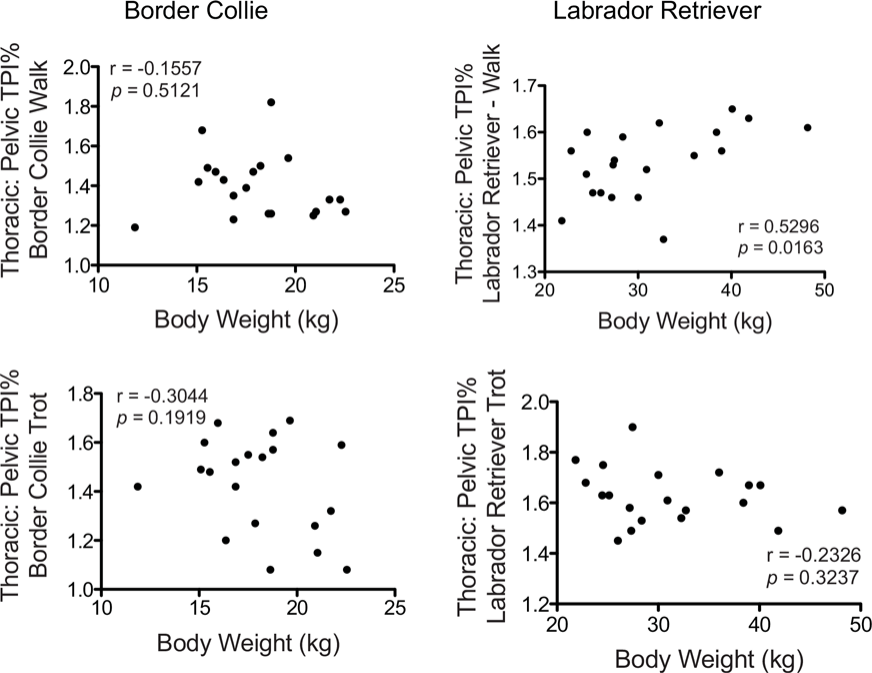
Thoracic to Pelvic Limb Total Pressure Index Percentage Ratio (T/P TPI%) compared to Body Weight at the Walk and Trot in Border Collies and Labrador Retrievers. There was no relationship between the ratio of thoracic to pelvic TPI% (T/P TPI%) and body weight at the trot for either breed (bottom left and bottom right) or for the Border Collies at the walk (top left). However, there was a significant positive correlation (p = 0.0163) between the ratio of weight borne on the thoracic limbs vs the pelvic limbs and body weight in the Labrador Retrievers at the walk (top right).

Because of the significant differences in the mean body weights between the Border Collies and the Labrador Retrievers, we examined whether there was a correlation between T/P TPI% ratios and body weight for both breeds at the walk and at the trot. There was no relationship between the T/P TPI% and body weight at the trot for either breed or for the Border Collies at the walk (Fig 3). However, there was a significant positive correlation (r = 0.5296; *p* = 0.0163) between the percentage of weight borne on the thoracic vs. the pelvic limbs and body weight in the Labrador Retrievers at the walk.

**Fig 4:**
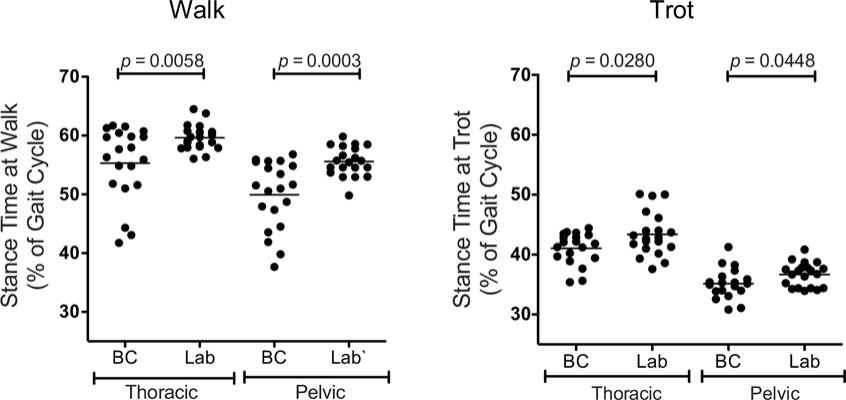
Stance Time as a Percentage of Gait Cycle (ST%) of the Thoracic and Pelvic Limbs at the Walk and Trot in Border Collies and Labrador Retrievers. Left. The Border Collies spent a significantly shorter proportion of the walking gait cycle with their thoracic and pelvic limbs in contact with the ground than the Labrador Retrievers (*p* < 0.0058 and *p* = 0.0003, respectively. Right. The Border Collies also spent a significantly shorter proportion of the trotting gait cycle with their thoracic and pelvic limbs in contact with the ground than the Labrador Retrievers (*p* < 0.0280 and *p* = 0.0448, respectively).

Stance Time Percentage (ST%) was also evaluated. The mean ST% for the thoracic limbs and pelvic limbs at the walk for the Border Collies were 55.3% and 49.9%, respectively, and for the Labrador Retrievers were 59.6% and 55.6%, respectively (Fig 4). The mean ST% of the thoracic and pelvic limbs at the trot for the Border Collies were 41.8% and 35.4%, respectively, and for the Labrador Retrievers were 43.5% and 36.7%, respectively (Fig 4). Thus, the Border Collies spent a significantly shorter proportion of both the walking and trotting gait cycles with their thoracic and pelvic limbs in contact with the ground than the Labrador Retrievers (walk: *p* < 0.0058 and *p* = 0.0003, for thoracic and pelvic limbs respectively, and trot: *p* < 0.0280 and *p* = 0.0448 for thoracic and pelvic limbs, respectively; Fig 4).

**Fig 5:**
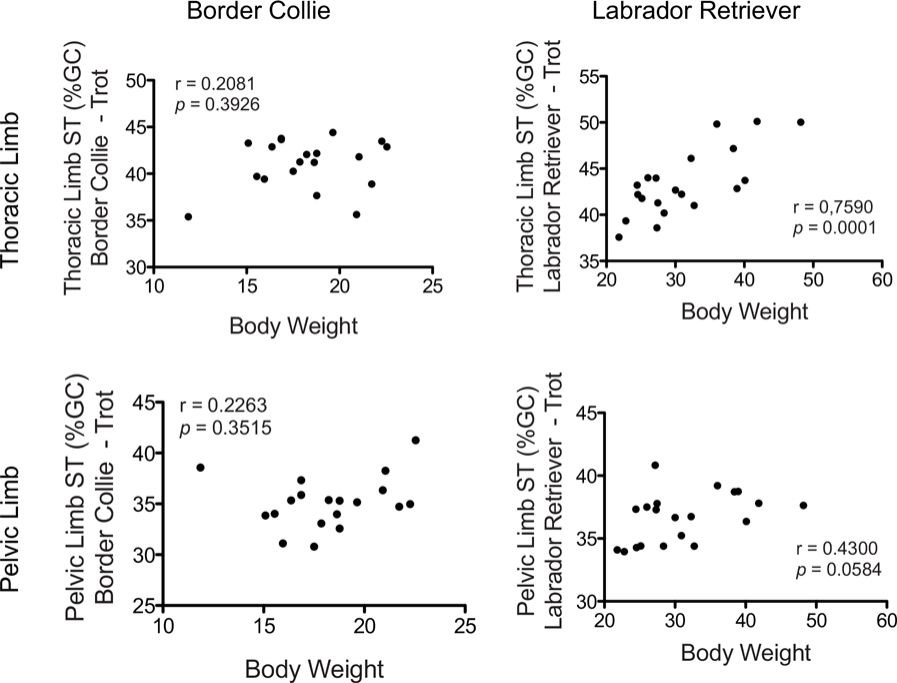
Thoracic and Pelvic Limb Stance Time as a Percentage of Gait Cycle (ST%) compared to Body Weight in Border Collies and Labrador Retrievers. There was no relationship between body weight and stance time of the thoracic or pelvic limbs in the trotting Border Collies. In the trotting Labrador Retrievers, there was a significant positive relationship between body weight and thoracic limb stance time and a strong trend towards a significant relationship between body weight and pelvic limb stance time.

To determine whether body weight affected ST% of the thoracic and/or pelvic limbs, we examined the relationship between body weight and thoracic or pelvic limb ST% in trotting Border Collies and Labrador Retrievers (Fig 5). There was no relationship between body weight and ST% of the thoracic or pelvic limbs in the trotting Border Collies.

In contrast, in trotting Labrador Retrievers, there was a significant positive relationship between body weight and thoracic limb ST% (r = 0.7590; *p* = 0.0001; Fig 5) and a strong trend towards a significant relationship between body weight and pelvic limb ST% (r = 0.4300; *p* = 0.0584).

**Fig 6:**
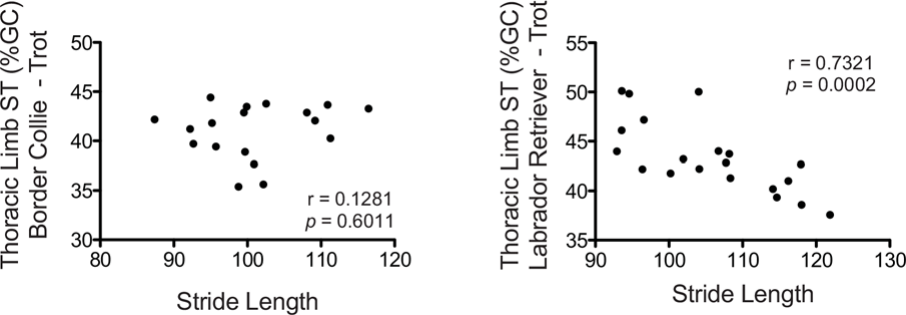
Thoracic Limb Stance Time as a Percentage of Gait Cycle (ST%) compared to Stride Length in Border Collies and Labrador Retrievers. As the Labrador Retrievers increased their stride lengths when trotting, they spent less time with their thoracic limbs in contact with the ground (r = 0.7321, p = 0.0002). In contrast, the amount of time spent with the thoracic limbs in contact with the ground did not change with stride length in the Border Collies.

Since it was determined that the Border Collies spent a lower percentage of the trotting gait cycle with their thoracic limbs in contact with the ground than the Labrador Retrievers, it was considered that this might be related to their shorter stride length (SrL) because of this breed’s smaller size. Thus, the relationship between thoracic limb ST% and SrL at the trot was examined. This was only examined for the thoracic limb because the SrL of both thoracic and pelvic limbs are similar. The Border Collies spent the same amount of time with their thoracic limbs in contact with the ground as their SrL increased (Fig 6). In contrast, the Labrador Retrievers with longer SrL spent less time with their thoracic limbs in contact with the ground (r = 0.7321, p = 0.0002; Fig 6).

Since the Border Collies spent a relatively shorter percentage of each gait cycle with their feet on the ground at both the walk and trot than the Labrador Retrievers, we questioned whether the Border Collies moved their feet faster over a given stride length. Thus, the stride times (SrT) for both the Border Collies and the Labrador Retrievers at the walk and the trot were examined.

**Fig 7:**
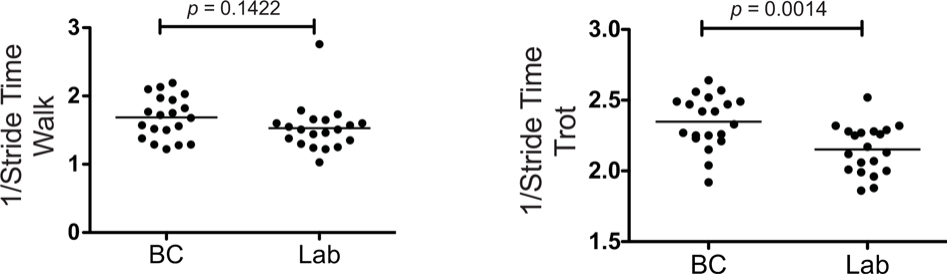
Stride Time at the Walk and Trot in Border Collies and Labrador Retrievers. There was no significant difference in the stride time between the two breeds at the walk. However, the Border Collies had a significantly lower stride time at the trot than the Labrador Retrievers.

While there was no significant difference in the stride time between the two breeds at the walk, the Border Collies had a significantly lower stride time at the trot than the Labrador Retrievers (*p* = 0.0014; Fig 7). Thus, when trotting, the Border Collies moved their feet faster at a given stride length than the Labrador Retrievers.

## Discussion

When analyzing a dog’s gait, it is important to consider that different breeds of dog scan have quite different conformation/structure as a result of having been bred for different original functions. As a result, it is possible that structurally dissimilar breeds might have significantly different quantitative gait characteristics. The Labrador Retriever, as the most populous breed in the United States, has classically been used as a “reference breed” for studies of gait [4,6,13–18,20,22]. We undertook the current study to determine whether there were significant differences in quantitative gait characteristics between Labrador Retrievers and Border Collies, another popular breed of dogs of similar size, but that is structurally dissimilar and that was bred for a different original function. In this report, we demonstrate several significant differences in gait between the two breeds. These differences should be taken into consideration when using quantitative gait analysis to identify lameness and diagnose musculoskeletal injuries in a clinical environment or when studying structure/gait relationships in a research setting.

Previous studies have shown that body weight, body condition, limb length, limb angulation, height at withers, and velocity affect ground reaction forces [5–11,17]. In this study the Border Collies placed significantly less pressure (TPI%) on the thoracic limbs relative to the pelvic limbs at both the walk and trot than the Labrador Retrievers. This was not a function of the Border Collies’ lighter body weight as there was no significant correlation between TPI% and body weight. These data indicate that the Border Collies bear proportionately less of their total body weight on the thoracic limbs than the Labrador Retrievers. The percentage of weight borne on the thoracic limbs of the Border Collies did not differ between the walk and trot, indicating that the Border Collie preserves its T/P TPI% regardless of overall body weight or velocity.

Thoracic limb TPI% in the Labrador Retrievers increased with increasing body weight in this breed at the walk and a strong positive trend at the trot, suggesting that greater thoracic limb TPI% was preserved in this breed. It is possible that the greater T/P TPI% in the Labrador Retriever is related to a larger and thus heavier head and forelimb musculature as an adaptation for carrying game [2,23].

The Border Collies spent a significantly shorter percentage of their gait cycle with their feet in contact with the ground at both a walk and a trot than the Labrador Retrievers. There was no correlation between body weight and the percentage of the gait cycle during which the thoracic or pelvic limbs were in contact with the ground for the Border Collies, suggesting that these animals have a shorter gait cycle, regardless of their weight. A shorter gait cycle could allow the Border Collies to have a higher stride frequency, which would allow this breed to move more quickly and change direction rapidly. Having a higher stride frequency to change direction quickly has been previously documented in dogs as well as horses and cheetahs [23–25].

In contrast, the Labrador Retrievers had a significant correlation between body weight and stance time for the thoracic limbs, and a strong trend towards a correlation between body weight and stance time for the pelvic limbs. This adaptation could enable a dog carrying heavy game to have a longer time with the feet in contact with the ground to drive through deep or tangled cover. This can be compared to weight pulling competitions in which dogs feet must have contact with the ground to pull the weight horizontally across the ground [23].

The stride time in the Border Collies and the Labrador Retrievers was also compared at the walk and the trot to determine whether the Border Collies move their feet faster over the ground than the Labrador Retrievers, which would contribute to a difference in ST% between the two breeds. While no significant difference was found at the walk, the Border Collies had a faster stride time ratio at the trot than the Labrador Retrievers. Thus, it appears that the Border Collies move their feet faster in relation to the distance traveled. Stride times also showed that the Border Collies take shorter steps when moving at a given speed. This could provide an advantage when turning because a dog that takes long steps will have its legs stretched further forward and rearward, which slows turning due to centrifugal forces produced by the extended limbs [23,24]. Ideally a dog should have its feet under the body when turning to reduce the amount of mass that is further from the center of rotation. This decreases centrifugal force, making it easier to turn [25].

Light et al previously published on temporal-spatial gait analysis variables by the use of a portable walkway system in healthy Labrador Retrievers at a walk and determined reference values for variables and symmetry ratios [6]. Labrador Retrievers were not evaluated at a trot in that study. Labrador Retrievers bore about 62% of their body weight on the thoracic limbs and 38% in the pelvic limbs when walking, and the average ratio of weight borne on the thoracic vs. pelvic limbs was 1.63. In the present study, the average percent body weight borne on the thoracic and pelvic limbs for the Labrador Retriever at the walk were 60.5% and 39.5%, respectively, resulting in a T/P TPI% of 1.53. The ST% of the thoracic and pelvic limbs for the Labrador Retriever at the walk were 55.55% and 50.25%, respectively, which is slightly lower than our finding of 59.5% and 55.6%, respectively. These differences may or may not be clinically significant; however, these discrepancies could be due to sample size or variations of morphology within the breed.

Limitations of this study include disadvantages intrinsic to using a pressure sensing walkway and temporospatial gait analysis system, including the inability to measure forces in three dimensions and thus, being able to quantitate only a product of total ground reaction force. Theoretically, either breed could be exerting craniocaudal forces that would not be detected from a simple vertical pressure analysis, which could affect their limb kinematics. Previous studies have used force plates to evaluate ground reaction forces of breeds with different structures and found differences in these forces [13–16]. Thus, data reported in this study are intrinsically different from studies using force plate analysis. However, previous studies were unable to provide both temporal and spatial information as collected from a pressure sensing walkway used in this study, which allowed for a multivariate approach to gait evaluation appropriate for the scope of this study [3–5].

Though objective gait analysis systems provide valuable quantitative information about gait characteristics, additional limitations include variation in the conformation and morphology not only between different breeds but also within them. Most of the dogs that participated in this study were well-conditioned performance dogs. This might not be true of all pets that are evaluated by gait analysis. Also, it is important to consider that there was a significant difference in body weight between the Border Collies and the Labrador Retrievers. While it may not be possible to find a healthy population of Border Collies and Labrador Retrievers with the same body weight range, it is important to note that the Border Collie had a narrower weight range than the Labrador Retriever. This may have placed the Border Collies at a disadvantage relative to the Labrador Retrievers when testing for correlation. Also, there was a slight (although not significantly different) difference in the velocities of the walk and the trot of the dogs in this study. While a potential clinical significance remains unknown, this may have affected gait parameters.

The significant differences in weight distribution and stance time between these two breeds should be considered when considering the function of each dog in a performance setting and their susceptibility to orthopedic injury. Further research is also needed determine the extent to which these reference values differ in dogs with various orthopedic disorders.

It is possible that the differences in quantitative gait characteristics identified in this study comparing Border Collies to Labrador Retrievers are related to differences in the original purposes for which these dogs were bred. Border Collies were originally bred for herding sheep, a job that required rapid changes in movement and speed. In contrast, Labrador Retrievers were bred for hunting, a job that required them to run straight to retrieve game and return directly to the hunter. These contrasting functions might lead to structural differences that would then be reflected in quantitative differences in gait characteristics for these two breeds. Likewise, there might be significant differences in quantitative gait characteristics between other breeds of dogs with differing functions (and therefore different structure) as well, such as the sight hounds (e.g. Whippets, Greyhounds and Borzoi), which were breed to chase small game using their vision over open fields, and the scent hounds (e.g. Basset Hounds, Beagles, and Foxhounds), which were bred to run with noses close to the ground as they follow the scent trails of their quarry.

Regardless of possible functional reasons for quantitative differences in dog gaits, this study suggests that breed-related differences in quantitative gait characteristics should be established and accounted for when assessing the gait of different breeds. Veterinarians assessing dogs for lameness or musculoskeletal injuries and researchers studying canine gait should be encouraged to take structural differences into account when quantitatively analyzing gait.

## Abbreviations

TPI%: total pressure index percentage for the thoracic and pelvic limbs
T/P TPI%: ratio of total pressure index percentage of the thoracic vs. pelvic limbs
ST%: stance time percentage for the thoracic and pelvic limbs
TSrL: stride length of the thoracic limbs
SrT: stride time

## References

[1] Border Collie Breed Standard. http://www.akc.org/breeds/border_collie/breed_standard.cfm last accessed April 24, 2014

[2] Labrador Retriever Breed Standard. http://www.akc.org/breeds/labrador_retriever/breed_standard.cfm last accessed April 24, 2014

[3] Quinn MM, Keuler NS, Lu Y, Faria ML, Muir P, Markel MD. Evaluation of agreement between numerical rating scales, visual analogue scoring scales, and force plate gait analysis in dogs. Vet Surg 2007; 36: 360–367. 17547599 10.1111/j.1532-950X.2007.00276.x

[4] Evans R, Horstman C, Conzemius M. Accuracy and optimization of force platform gait analysis in Labradors with cranial cruciate disease evaluated at a walking gait. Vet Surg 2005; 34:445–449. 16266335 10.1111/j.1532-950X.2005.00067.x

[5] Voss K, Imhof J, Kaestner S, Montavon PM. Force plate gait analysis at the walk and trot in dogs with low-grade hindlimb lameness. Vet Comp Orthop Traumatol 2007; 20: 299–304. 18038008 10.1160/vcot-07-01-0008

[6] Light VA, Steiss JE, Montgomery RD, Rumph PF, Wright JC. Temporal-spatial gait analysis by the use of a portable walkway system in healthy Labrador Retrievers at a walk. Am J Vet Res 2010; 71:997–1002. 10.2460/ajvr.71.9.997 20807137

[7] Kim J, Kazmierczak KA, Breur GJ. Comparison of temporospatial and kinetic variables of walking in small and large dogs on a pressure-sensing walkway. Am J Vet Res 2011; 72:1171–1177. 10.2460/ajvr.72.9.1171 21879974

[8] Lasceles BD, Roe SC, Smith E, Reynolds L, Markham J, Marcellin-Little D, et al Evaluation of a pressure walkway system for measurement of vertical limb forces in clinically normal dogs. Am J Vet Res 2006; 67:277–282. 16454633 10.2460/ajvr.67.2.277

[9] Besancon MF, Conzemius MG, Derrick TR, Ritter MJ. Comparison of vertical forces in normal greyhounds between force platform and pressure walkway measurement systems. Vet Comp Orthop Traumatol 2003; 16:153–157.

[10] Moreau M, Troncy E, Bichot S, Lussier B. Influences of changes in body weight on peak vertical force in osteoarthritic dogs: a possible bias in study outcome. Vet Surg 2010; 39: 43–47. 10.1111/j.1532-950X.2009.00621.x 20210943

[11] Brady RB, Sidiropoulos AN, Bennett HJ, Rider PM, Marcellin-Little DJ, Devita P. Evaluation of gait-related variables in lean and obese dogs at a trot. Am J Vet Res 2013;74:757–762. 10.2460/ajvr.74.5.757 23627389

[12] Gillette RL, Angle TC. Recent developments in canine locomotor analysis: a review. The Vet J 2008; 178: 165–176. 10.1016/j.tvjl.2008.01.009 18406641

[13] Bertram JEA, Lee DV, Case HN, Todhunter RJ. Comparison of the trotting gaits of Labrador Retrievers and Greyhounds. Am J Vet Res 2000; 61: 832–838. 10895909 10.2460/ajvr.2000.61.832

[14] Colborne GR, Innes JF, Comerford EJ, Owen MR, Fuller CJ. Distribution of power across the hindlimbs in Labrador Retrievers and Greyhounds. Am J Vet Res 2005; 66: 1563–1571. 16261830 10.2460/ajvr.2005.66.1563

[15] Besancon MF, Conzemius MG, Evans RB, Ritter MJ. Distribution of vertical forces in the pads of Greyhounds and Labrador Retrievers during walking. Am J Vet Res 2004;65:1497–1501. 15566087 10.2460/ajvr.2004.65.1497

[16] Molsa SH, Hielm-Bjorkman AK, Laitinen-Vapaavuori. Force platform analysis in clinically healthy Rottweilers: comparison with Labrador Retrievers. Vet Surg 2010; 39: 701–707. 10.1111/j.1532-950X.2010.00651.x 20345537

[17] Voss K, Galeandro L, Wiestner T, Haessig M, Montavon PM. Relationships of body weight, body size, subject velocity, and vertical ground reaction forces in trotting dogs. Vet Surg 2010; 39:863–869. 10.1111/j.1532-950X.2010.00729.x 20825596

[18] Nordquist B, Fischer J, Kim SY, Stover SM, Garcia-Nolen T, Hayashi K, et al Effects of trial repetition, limb side, intraday and inter-week variation on vertical and craniocaudal ground reaction forces in clinically normal Labrador Retrievers. Vet Comp Orthop Traumatol 2011; 24: 435–444. 10.3415/VCOT-11-01-0015 21938309

[19] Torres BT, Moëns NM, Al-Nadaf S, Reynolds LR, Fu YC, Budsberg SC. Comparison of overground and treadmill-based gaits of dogs. Am J Vet Res 2013; 74: 535–541. 10.2460/ajvr.74.4.535 23531060

[20] Agostinho FS, Rahal SC, Miqueleto NS, Verdugo MR, Inamassu LR, El-Warrak AO. Kinematic analysis of Labrador retrievers and rottweilers trotting on a treadmill. Vet Comp Orthop Traumatol 2011; 24: 185–191. 10.3415/VCOT-10-03-0039 21327291

[21] Nunamaker DM, Blauner PD. Normal and abnormal gait In Newton CD, Nunamaker DM, eds. Textbook of small animal orthopaedics. Philadelphia: JB Lippincott Co, 1985; 1084–1085.

[22] Smith Stephen. Most popular dog breed in America. American Kennel Club. 2015;2: Available: http://www.akc.org/news/the-most-popular-dog-breeds-in-america/

[23] Brown, Curtis M., and Bonnie Dalzell. *Dog Locomotion and Gait Analysis*. Wheat Ridge, Colo., U.S.A. (4401 Zephyr St., Wheat Ridge 80033–3299): Hoflin Pub., 1986. Print.

[24] Betram JEA and Gutmann A. Motions of the running horse and cheetah revisited: fundamental mechanics of the transverse and rotary gallop. J R Soc Interface 2009; 6: 549–559. 10.1098/rsif.2008.0328 18854295 PMC2696142

[25] Hudson PE, Corr SA, Wilson AM. High speed galloping in the cheetah (*Acinonyx jubatus*) and the racing greyhound (*Canis familiaris*): spatio-temporal and kinetic characteristics. J Exp Biol 2012 215; 2425–2434. 10.1242/jeb.066720 22723482

